# Vitamin D increases glucocorticoid efficacy via inhibition of mTORC1 in experimental models of multiple sclerosis

**DOI:** 10.1007/s00401-019-02018-8

**Published:** 2019-04-27

**Authors:** Robert Hoepner, Maud Bagnoud, Maximilian Pistor, Anke Salmen, Myriam Briner, Helen Synn, Lisa Schrewe, Kirsten Guse, Farhad Ahmadi, Seray Demir, Louis Laverick, Melissa Gresle, Paul Worley, Holger Michael Reichardt, Helmut Butzkueven, Ralf Gold, Imke Metz, Fred Lühder, Andrew Chan

**Affiliations:** 1 Department of Neurology, Inselspital Bern, University Hospital, University of Bern, Bern, Switzerland; 2 Department of Neurology, St. Josef Hospital, Ruhr University Bochum, Bochum, Germany; 3 Institute of Neuropathology, University Medical Center Goettingen, Goettingen, Germany; 4 Department of Medicine, Royal Melbourne Hospital, University of Melbourne, Melbourne, Australia; 5 The Solomon H. Snyder Department of Neuroscience, School of Medicine, John Hopkins University, Baltimore, USA; 6 Institute for Cellular and Molecular Immunology, University Medical Center Goettingen, Georg-August-University Goettingen, Goettingen, Germany; 7 Department of Neuroscience, Central Clinical School, Alfred Campus, Monash University, Melbourne, Australia; 8 Institute of Neuroimmunology and Multiple Sclerosis Research, University Medical Center Goettingen, Georg-August-University Goettingen, Goettingen, Germany

**Keywords:** Mammalian target of rapamycin, Steroid resistance, Calcitriol, c-Jun N-terminal kinase, Relapse treatment

## Abstract

The limited efficacy of glucocorticoids (GCs) during therapy of acute relapses in multiple sclerosis (MS) leads to long-term disability. We investigated the potential of vitamin D (VD) to enhance GC efficacy and the mechanisms underlying this VD/GC interaction. In vitro, GC receptor (GR) expression levels were quantified by ELISA and induction of T cell apoptosis served as a functional readout to assess synergistic 1,25(OH)_2_D_3_ (1,25D)/GC effects. Experimental autoimmune encephalomyelitis (MOG_35–55_ EAE) was induced in mice with T cell-specific GR or mTORc1 deficiency. 25(OH)D (25D) levels were determined in two independent cohorts of MS patients with stable disease or relapses either responsive or resistant to GC treatment (initial cohort: *n* = 110; validation cohort: *n* = 85). Gene expression of human CD8^+^ T cells was analyzed by microarray (*n* = 112) and correlated with 25D serum levels. In vitro, 1,25D upregulated GR protein levels, leading to increased GC-induced T cell apoptosis. 1,25D/GC combination therapy ameliorated clinical EAE course more efficiently than respective monotherapies, which was dependent on GR expression in T cells. In MS patients from two independent cohorts, 25D deficiency was associated with GC-resistant relapses. Mechanistic studies revealed that synergistic 1,25D/GC effects on apoptosis induction were mediated by the mTOR but not JNK pathway. In line, 1,25D inhibited mTORc1 activity in murine T cells, and low 25D levels in humans were associated with a reduced expression of mTORc1 inhibiting tuberous sclerosis complex 1 in CD8^+^ T cells. GR upregulation by 1,25D and 1,25D/GC synergism in vitro and therapeutic efficacy in vivo were abolished in animals with a T cell-specific mTORc1 deficiency. Specific inhibition of mTORc1 by everolimus increased the efficacy of GC in EAE. 1,25D augments GC-mediated effects in vitro and in vivo in a T cell-specific, GR-dependent manner via mTORc1 inhibition. These data may have implications for improvement of anti-inflammatory GC therapy.

**Electronic supplementary material:**

The online version of this article (10.1007/s00401-019-02018-8) contains supplementary material, which is available to authorized users.

## Introduction

Multiple sclerosis (MS) is an autoimmune disorder of the central nervous system. At disease onset, more than 80% of MS patients suffer from a relapsing disease course [15]. MS relapses are characterized by acute occurrence of new neurological symptoms or worsening of pre-existing deficits due to acute focal autoimmune inflammation. Relapses often lead to sustained residual disability with almost 50% of patients suffering increased disability post-relapse [26]. High-dose intravenous glucocorticoid (GC) pulse therapy, i.e. 500–2000 mg methylprednisolone (MP) over 3–5 days, is the current standard treatment of acute MS relapses. A plethora of experimental data indicates that termination of the autoimmune inflammatory reaction by GCs attenuates tissue damage [27]. Several mechanisms are considered to contribute to these profound anti-inflammatory effects, e.g. inhibition of pro-inflammatory mediators, inhibition of activation and migration of immune cells, and restoring integrity of the blood–brain barrier [27, 46]. Induction of T cell apoptosis in both MS patients and experimental autoimmune encephalomyelitis (EAE), an animal model that mimics many features of MS, is also considered to contribute to the therapeutic mechanism of GCs [23, 44]. In EAE, GC efficacy is dependent on the presence of the glucocorticoid receptor (GR) expressed in T cells [52, 54]. Although GCs constitute the mainstay of relapse therapy in MS, a considerable proportion of patients do not respond sufficiently [6]. This impaired clinical efficacy has been linked to altered levels of the GR and associated proteins [2]. Namely, reduced expression of the GR in peripheral blood mononuclear cells of GC-resistant MS patients was accompanied by increased levels of the chaperone heat shock protein (hsp) 90 [29]. In addition, a downregulation of the GR was detected in GC-resistant T cells obtained during myelin oligodendrocyte glycoprotein_35–55_ (MOG_35–55_) EAE [14]. Clinically, overcoming GC resistance remains an unmet need in MS therapy. Several lines of evidence indicate that vitamin D (VD) could augment GC-induced immune effects. For instance, VD pre-treatment increased dexamethasone-induced binding of the GR to GC response elements in monocytes from patients with GC-resistant asthma [57]. Furthermore, VD restored the efficacy of GCs to induce IL-10 production in T-helper cells [55]. Hypothetically, the inhibition of mammalian target of rapamycin (mTOR) by VD could mediate such a synergistic effect, since in malignant lymphoid cells in vitro resistance to GCs appears to be reversible after application of the mTOR inhibitor rapamycin [31]. The mTOR pathway controls cell differentiation and survival by integrating multiple environmental cues such as stress, oxygen supply, growth factors and amino acids. In particular, mTORc1 regulates cell metabolism and growth, whereas mTORc2 affects cell proliferation and survival in a cell-type-specific manner [42]. Inhibition of mTOR by VD was previously described in osteoblasts [25] and more recently it was also demonstrated that VD administration downregulates mTOR in CD4^+^ T cells during rat EAE [56]. As an alternative pathway, c-Jun N-terminal kinases (JNK) pathway has also been implicated in the regulation of GR expression [51]. JNK belongs to a highly preserved ubiquitous signaling pathway, the mitogen-activated protein (MAP) kinases [41] and activation of JNK was found to be associated with a poor response to GCs in patients with Crohn’s disease [41]. Here, we set out to investigate the potential synergistic effects of VD and GCs in vitro, in EAE and in MS patients in the context of acute relapses, and to unravel the cellular and molecular mechanisms underlying the effects of VD on GC therapy. We found that VD increases the efficacy of GC treatment by upregulating the GR via mTORc1 in T cells but not by JNK signaling, which suggests that combination therapy with mTOR inhibiting substances bears the potential to overcome GC resistance in the treatment of MS relapses.

## Materials and methods

The active metabolite 1,25(OH)_2_D_3_ (1,25D) was used in all in vitro and in vivo studies. Further, it was measured in murine serum samples to assess bioavailability after oral gavage of 1,25D. For human serum concentrations, 25-hydroxyvitamin D (25D) was analyzed as it represents the body’s VD supply [18].

### Studies with human samples

Human studies were approved by the cantonal ethics committee Bern and the local ethics committee of Ruhr-University Bochum and Goettingen (5408-15, 4801-13, 2017-00060, 2017-01369, E08-910, 19-09-10). CD3^+^ T cells were isolated using gradient centrifugation and negative magnetic cell sorting (Pan T cell isolation kit^®^, Miltenyi Biotec, Cologne, Germany). 1 × 10^6^/ml T cells (RPMI, Invitrogen, Karlsruhe, Germany; 10% FCS, Sigma-Aldrich, St. Louis, USA; 1% Penicillin/Streptomycin (PenStrep), Invitrogen; l-Glutamine, Invitrogen, Sigma-Aldrich) were stimulated with phytohemagglutinin (PHA, 0.5 µg/ml, Sigma-Aldrich) and treated with control (solvent: 0.1% DMSO), 10 nM 1,25D (MedChem Express, South Brunswick, New Jersey, USA) or 75 µM methylprednisolone (MP, MIBE GmbH, Brehna, Germany) or the combination of 1,25D/MP (37 °C, 5% CO_2_). Furthermore, the mTOR inhibitors rapamycin (5.5 nM, Millipore, Darmstadt, Germany), voxtalisib (1.57 µM, MedChemExpress), and everolimus (2 nM, Seleck Chemicals, Houston, Texas USA) as well as the JNK inhibitor SP600125 (10 nM, MedChem Express) were used alone or in combination with 75 µM MP. T cell apoptosis was analyzed by flow cytometry (FACS) after 24 or 72 h (annexin V/propidium iodide, Becton–Dickinson Bioscience, Franklin Lakes, New Jersey, USA). For the analysis of GR expression, CD3^+^ T cells (1 × 10^6^/ml) from healthy donors (HD, *n* = 6) were seeded in a poly-l-lysine-coated 96-well plate (10 µg/ml, Sigma-Aldrich). Cells were treated with control (solvent: 0.1% DMSO) or 1,25D for 24 h (10 nM, 100 nM; 37 °C, 5% CO_2_), and the GR was quantified by ELISA according to the manufacturer’s protocol (NR3C1 Human Cell-Based ELISA kit, Abnova, Taipei, Taiwan). Data are expressed as ratio of optic density (OD)_450_ GR/glyceraldehyde 3-phosphate dehydrogenase (GAPDH).

To test whether the GR is differentially regulated in different T cell subsets, human GR was analyzed by western blot in CD4^+^ and CD8^+^ T cells. Cells were isolated from frozen PBMC collected after GC administration (negative selection; CD4^+^ T Cell Isolation Kit, human; CD8^+^ T Cell Isolation Kit, human; Miltenyi Biotec; Germany). After lysis (10% trichloroacetic acid buffer, Fluka, Switzerland), western blots were stained with following antibodies and analyzed with Fusion Imaging System (Vilber Lourmat, France): Anti-GR, 1:50,000 (abcam, United Kingdom); Anti-HSP90, 1:1000 (Cell Signaling, Massachusetts); Anti-GAPDH, 1:10,000 (abcam, United Kingdom); Goat Anti-Rabbit IgG (H + L) HRP, 1:3000 (Bio-Rad, California). In addition, early active demyelinating MS lesions, obtained via stereotactic biopsy before GC administration, were co-stained for GR and either CD3, CD4 or CD8.  The following antibodies were used for staining of paraffin slices: Anti-GR, 1:300 (Cell Signaling Technology); Anti-CD3, 1:50 (Zytomed System, Berlin, Germany); Anti-CD4, 1:100 (Zytomed System); Anti-CD8, 1:250 (CellMarque, Rocklin, USA) [30].

A retrospective chart analysis identified stable MS patients (initial cohort; University of Bochum, *n* = 56, McDonald criteria; relapsing phenotype, no relapse or intravenous GCs within the last 3 months) and MS patients during acute relapse (initial cohort: *n* = 54; < 30 days since symptom onset). An independent validation cohort included *n* = 85 patients (stable MS, *n* = 40; MS relapse, *n* = 45, University of Bern). Relapse definition followed standard criteria [13]. Patients were defined as GC responsive if they no longer showed relapse-associated neurological symptoms within 2 months from relapse onset (initial cohort *n* = 30; validation cohort *n* = 20) [17]. In line with current guidelines [13], patients with insufficient clinical response as defined by clinical experts (e.g. visual acuity < 0.5 or EDSS pyramidal functional subscore > 3) to ≥ 2 high-dose GC pulses (e.g. 500–2000 mg MP over 3–5 days) underwent treatment intensification with plasma exchange within 6 weeks after relapse onset. These patients were classified as GC resistant (initial cohort *n* = 24; validation cohort *n* = 25) [43].

Serum levels of 25D, which represent the body’s VD supply, were analyzed by electro-chemiluminescence binding assay (initial cohort: Elecsys^®^, Roche, Basel, Switzerland). For analysis of 25D and 1,25D serum levels in the independent validation cohort, Liason^®^ 25D Total Assay and 1,25D Assay were used (DiaSorin Inc, Stillwater, USA).

For gene expression analyses, CD8^+^ T cells (from 63 healthy donors and 49 MS patients) were isolated using gradient centrifugation and magnetic cell sorting (Miltenyi Biotec). A WT expression kit (Life technologies, Carlsbad, California, USA) was used to generate and amplify sense strand DNA for fragmentation and labeling using a Gene Chip WT terminal labelling kit (Affymetrix, Santa Clara, California, USA). Samples were hybridized to Affymetrix Human gene 1.0 ST chips (Affymetrix), containing 764,885 distinct oligonucleotide features consolidated into 33,297 probe sets. The Bioconductor Affymetrix package was then used to perform robust multichip average computations. Unwanted variation from the expression data was removed using the remove unwanted variation two-step method.

### Animal studies

Animal studies were approved by the local authorities (Office of Agriculture and Nature Bern, Switzerland; no. BE 101/16; Regional Office for Nature, Environment and Consumer Protection of North Rhine-Westphalia, Germany: no. 84-02.04.2015.A051). Mice were bred under conventional housing conditions at the in-house animal facilities of the University of Bern, the Ruhr University Bochum and the University Medical Center Goettingen. In addition to C57BL/6 and BALB/c wild-type (WT) mice (Janvier Labs, Le Genest-Saint-Isle, France), we also used animals with altered GR signaling: GR^wt^ (Nr3c1) and GR^dim^ (Nr3c1^tm3GSc^) mice on a BALB/c background; GR^flox^ (Nr3c1^tm2GSc^) and GR^lck^ (Nr3c1^tm2GSc^Tg^(lck-cre)1Cwi^) mice on a C57BL/6 background [7, 25]. GR^lck^ mice do not express the GR specifically in T cells [7], whereas a point mutation present in GR^dim^ mice impairs GR-mediated effects due to impaired dimerization of the receptor in all cell types. Other GR signaling pathways are preserved in GR^dim^ mice [37]. C57BL/6 Rheb^lck^ (mTORc1 KO) has a T cell-specific depletion of mTORc1 activity as described previously [9].

Splenic CD3^+^ T cells were isolated from WT, GR^dim^ and Rheb^lck^ mice by magnetic cell sorting (Pan T cell isolation kit^®^, Miltenyi Biotec). 1 × 10^6^/ml T cells [RPMI media, FCS 10%, PenStrep 1%, l-glutamine 1% (Sigma-Aldrich)] were stimulated with concanavalin A (ConA, 1.5ug/ml, Sigma-Aldrich) and treated with control (solvent: 0.1% DMSO), 1,25D (10 nM and 100 nM) and MP (6–600 nM, 24–48 h, 37 °C, 5% CO_2_). The viability of these T cells was analyzed by FACS (annexin V/propidium iodide staining). For the analysis of mTOR inhibition, T cells were treated with different concentrations of 1,25D (10 and 100 nM) or rapamycin (547 pM) in vitro (24 h). After lysis (HEPES 50 mM, NaCl 150 mM, glycerol 10%, EDTA 1 mM, Nonidet P-40, 1%), western blot was performed to determine mTORc1 activity (Phospho-p70 S6 Kinase (phP70S6K), Cell Signaling Technology, Danvers, USA, 1:500; beta-Actin, Sigma-Aldrich, 1:15,000). Protein expression is presented as the densitometric ratio of target to housekeeping protein (Image Studio 4.0, LI-COR, Lincoln, USA). To test GR regulation by 1,25D in regard to mTORc1 signaling, T cells from WT and Rheb^lck^ mice were treated in vitro for 24 h with control (solvent: 0.1% DMSO) or 1,25D (10 nM). GR concentration was measured using a commercial available ELISA (Mouse GR ELISA kit; MyBioSource, San Diego, USA). For EAE experiments WT, GR^lck^ as well as Rheb^lck^ on C57Bl6 background mice were used. Induction of MOG_35–55_ EAE followed our previously described protocols [45]. Briefly, animals were immunized by a subcutaneous injection of 100 µg MOG_35–55_ peptide (Institute of Medical Immunology, Charité, Berlin, Germany) in PBS (Thermofischer, Dreieich, Germany) emulsified in complete Freund’s adjuvant containing 100 µg *Mycobacterium tuberculosis* H37RA (Difco, Detroit, Michigan, USA), followed by 200 ng pertussis toxin (Quadratech, Epsom, United Kingdom) (i.p., days 0 and 2). Animals were scored daily in a blinded manner using a 10-point EAE scale: 0, normal; 1, reduced tone of tail; 2, limp tail, impaired righting; 3, absent righting; 4, gait ataxia; 5, mild paraparesis of hind limbs; 6, moderate paraparesis; 7, severe paraparesis or paraplegia; 8, tetraparesis; 9, moribund; 10, death [45]. Treatment was initiated when ≥ 50% of the animals had an EAE score ≥ 2.

MP (0.8 mg/kg/day or 4 mg/kg/day i.p.), 1,25D (10 ng/day, oral gavage), everolimus (5 mg/kg/day, oral gavage), SP600125 (15 mg/kg/day, oral gavage) and controls (solvents for MP: PBS i.p.; for 1,25D/everolimus/SP600125: DMSO in peanut oil p.o., Byodo, Mühldorf am Inn, Germany) were given on three consecutive days. Due to dual drug administration in the combination therapy arm, the control group received two control substances (solvents PBS i.p. and DMSO (in peanut oil p.o.). Further, each monotherapy arm received the control substance of the respective other drug (e.g. MP monotherapy: MP in PBS and DMSO in peanut oil p.o. as 1,25D/everolimus control substance).

For the analysis of the bioavailability of oral 1,25D used during EAE experiments, we determined peak serum level of 1,25D. For this purpose, 1,25D (10 ng/day) or control (solvent: DMSO in peanut oil) was fed via oral gavage over three consecutive days and blood was collected on day 3, 4 h after last 1,25D administration. Murine 1,25D serum levels were determined using a chemiluminescence immunoassay (LIAISON^®^, DiaSorin, Stillwater, MN, USA).

### Statistical analysis

All statistical analyses were performed with SPSS^®^ 20 (IBM Corporation, Armonk New York, USA). Spearman’s Rho was used for correlation analysis. The effect of mTOR/JNK inhibitor or 1,25D on MP-induced T cell apoptosis (human/murine) and GR protein expression was evaluated by a Wilcoxon signed-rank test. Further, Mann–Whitney test was applied for comparison of murine 1,25D serum concentrations and ex vivo apoptosis in animals fed with 1,25D or control. For comparison of categorical data sets, Chi-square test was run. Kruskal–Wallis test adjusted for multiple comparisons using Bonferroni correction was performed for analysis of EAE disease course, 25D serum concentration and gene expression of key regulators of mTORc1 activity. Further, a multivariate logistic regression analysis was used to calculate the risk of a GC-resistant relapse in MS patients with severe 25D deficiency.

## Results

### 1,25D increases glucocorticoid-induced apoptosis of human and murine T cells via upregulation of the glucocorticoid receptor

**Fig. 1:**
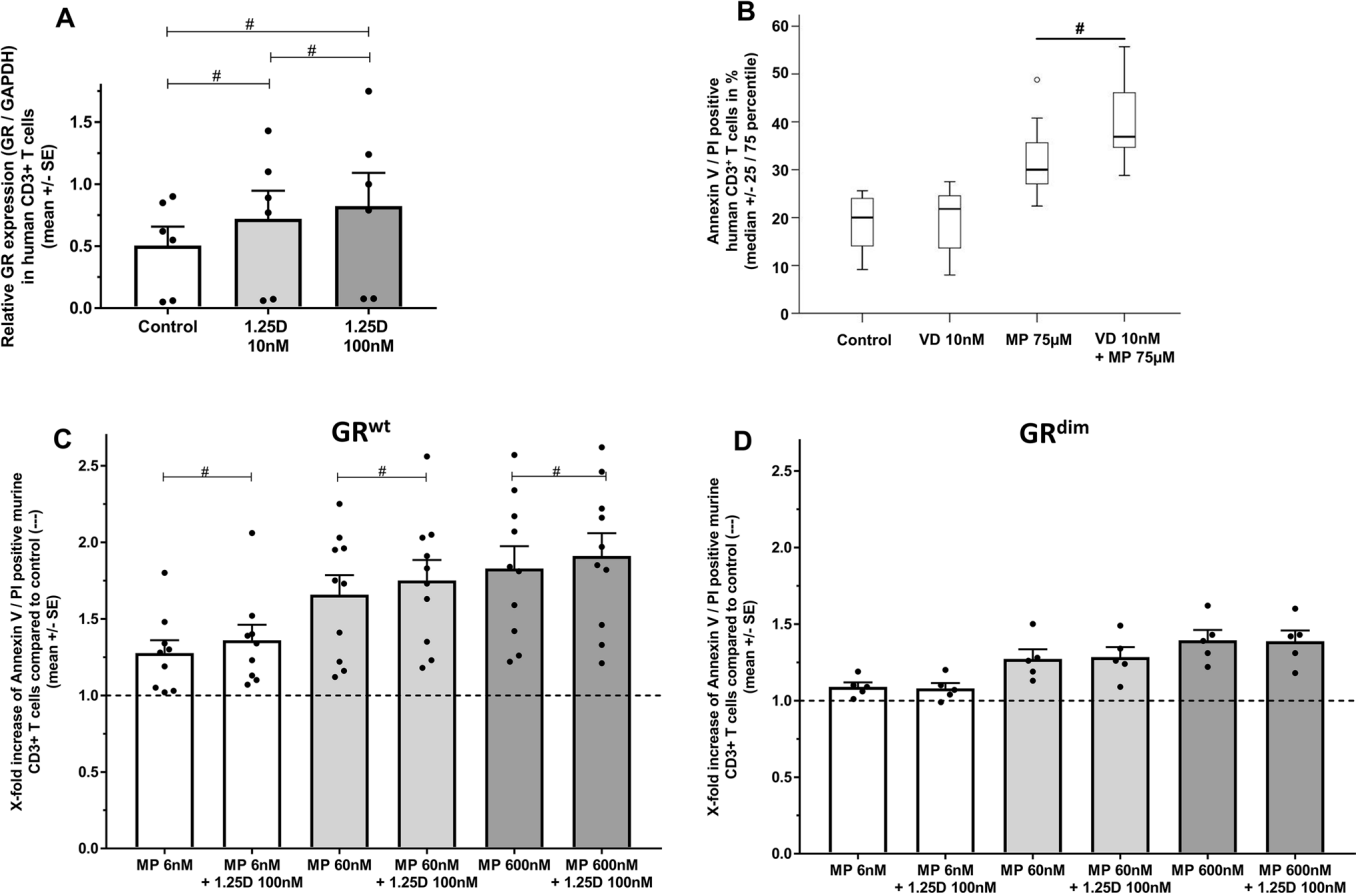
**a** GR protein expression normalized to glyceraldehyde 3-phosphate dehydrogenase (GAPDH) after incubation of human CD3^+^ T cells with 1,25D in vitro (24 h; PHA 0.5 µg/ml; *n* = 6 per group. Cell-based ELISA). Data are expressed as ratio of optical density (OD) 450 GR/GAPDH. **b** Apoptosis of human CD3^+^ T cells of healthy donors. Incubation with control (solvent: 0.1% DMSO), 1,25D, MP or 1,25D + MP in vitro (72 h; PHA 0.5 µg/ml; *n* = 7 per group; Annexin V/PI, flow cytometry). Apoptosis of murine splenic-derived CD3^+^ T cells from **c** BALB/c GR^wt^ (wild type) or **d** BALB/c GR^dim^ mice. Incubation with control (solvent: 0.1% DMSO), 1,25D, MP or 1,25D + MP in vitro (24 h; ConA 1.5 µg/ml; *n* = 9–10 (GR^wt^); *n* = 5 (GR^dim^); Annexin V/PI, flow cytometry). Apoptosis is depicted as increase over control (–; control: solvent 0.1% DMSO). In the control groups of each genotype, mean percentage of apoptotic murine T cells (SE) was as follows: BALB/c GR^wt^ 46.1% (4.3) vs BALB/c GR^dim^ 42.3% (3.6). *GR* glucocorticoid receptor, *OD* optical density, *1,25D* 1,25(OH)_2_D_3_, *MP* methylprednisolone, *SE* standard error. Statistics: Wilcoxon signed-rank test: # < 0.05

Given the proposed importance of the expression level of the GR in clinical GC resistance, we first investigated whether 1,25D modifies GR protein levels in human T cells. As depicted in Fig. 1a, 1,25D (10 and 100 nM) increased GR expression in a dose-dependent manner in vitro. In line with this observation, GR gene expression in CD8^+^ T cells from a different cohort demonstrated a moderate reduction in individuals with severe 25D deficiency (mean (SE) log2 GR gene expression: serum 25D level: < 25 nmol/l: 9.94 (0.14), *n* = 5 (*n* = 2 healthy controls/*n* = 3 stable MS patients); ≥ 25 nmol/l: 10.09 (0.14), *n* = 107 (*n* = 61 healthy controls/*n* = 46 stable MS), *p* < 0.05, Affymetrix human gene 1.0 ST chip). To address the functional relevance of 1,25D-induced GR upregulation, we employed the GR-dependent model of GC-induced CD3^+^ T cell apoptosis in vitro. Using a concentration of 75 µM methylprednisolone (MP), which is achieved by intravenous pulse therapy during treatment of MS relapses [1], co-incubation with 1,25D (10 nM) led to a higher apoptosis rate of human T cells compared to MP alone (Fig. 1b). We next examined whether the synergistic 1,25D/MP effects that we observed in vitro could also be detected in mice ex vivo. For this purpose, C57BL/6 WT mice were fed with 1,25D or control (control: DMSO in peanut oil) on three consecutive days before splenic T cells were isolated and incubated in vitro for 24 h with MP. Pre-treatment of animals with 1,25D led to increased GC-induced apoptosis of murine T cells over a broad MP dose range (1.1–1.3-fold, *p* < 0.01, Online Resource 1). To test whether the observed synergistic effect of 1,25D on GC-induced T cell apoptosis was mediated via the GR, we utilized rodent cells with a functional impairment of the GR (GR^dim^). These cells harbor a point mutation that compromises GR dimerization; however, other GR signaling pathways are preserved, which explains residual effects of GCs to induce apoptosis in GR^dim^ T cells [37]. A synergistic effect of 1,25D and MP on the induction of T cell apoptosis was observed in T cells with a functional GR (GR^wt^, Fig. 1c), whereas this effect was not detectable in GR^dim^ T cells (Fig. 1d).

### 1,25D augments therapeutic efficacy of methylprednisolone in MOG_35–55_ experimental autoimmune encephalomyelitis in a glucocorticoid receptor-dependent manner

**Fig. 2:**
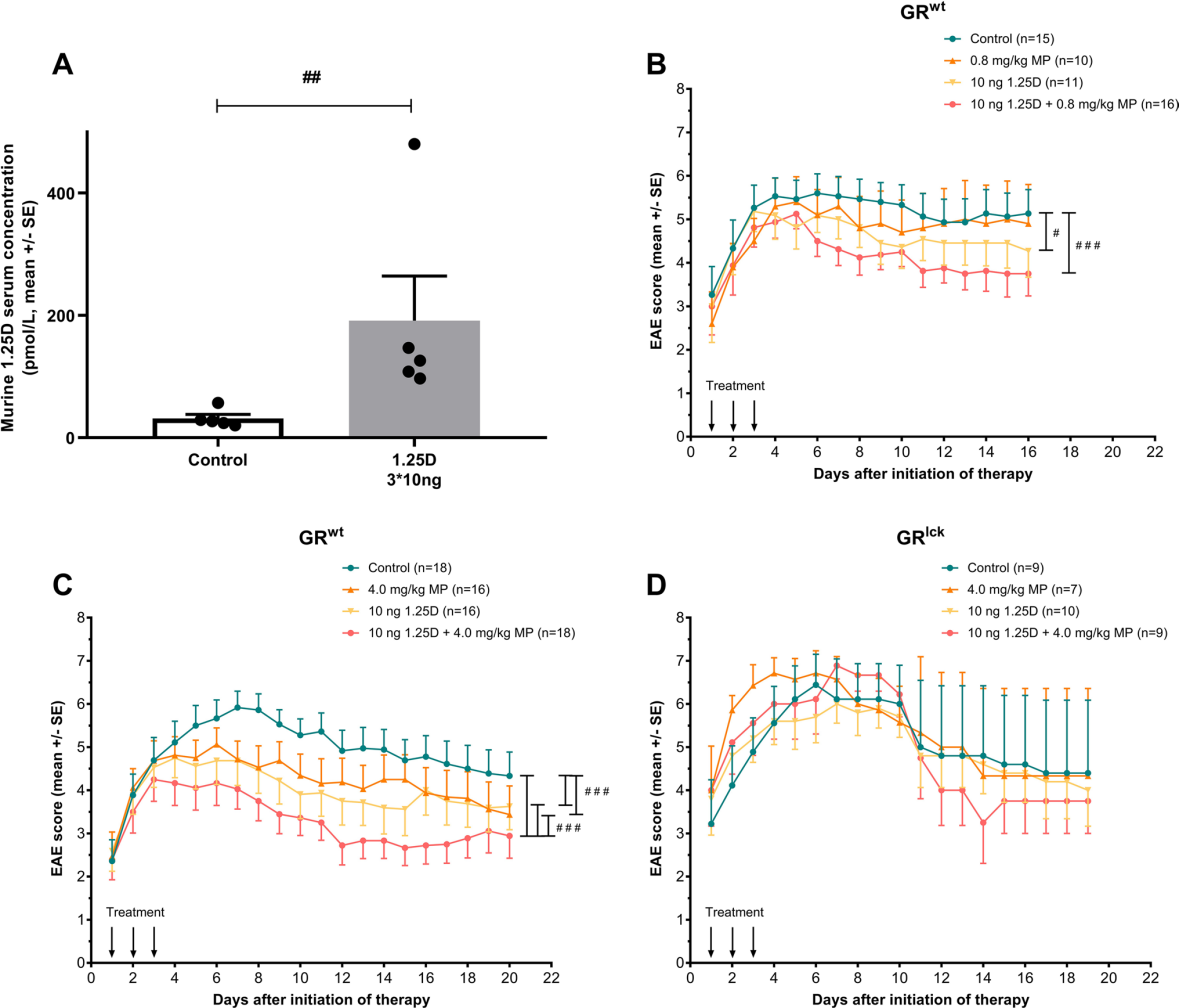
**a** Murine 1,25D serum concentration after 3 days of oral gavage with 1,25D 10 ng/day or control (solvent: DMSO in peanut oil). Cardiac blood was sampled 4 h after the last oral gavage (*n* = 5 per group, chemiluminescence immunoassay). Clinical disease course of MOG_35–55_ EAE in C57BL/6 wild-type mice **b**, **c** or GR^lck^ mice devoid of the T cell GR **d** treated for three consecutive days with control (DMSO in peanut oil), 1,25D (10 ng/day), MP [**b** intermediate MP dosage (0.8 mg/kg/day), **c**, **d** high MP dosage (4.0 mg/kg/day)] or 1,25D + MP. Downwards arrow: days of treatment. EAE score: 10-score system according to Ref. [45]. Numbers of included animals are given in the figure. *EAE* experimental autoimmune encephalomyelitis, *GR*^*lck*^ mice with glucocorticoid receptor deficient T cells, *GR*^*wt*^ glucocorticoid receptor wild type, *MP* methylprednisolone, *SE* standard error, *1,25D* 1,25(OH)_2_D_3_. Statistics: **a** Mann–Whitney test: ## < 0.01, **b**–**d** Kruskal–Wallis test: # < 0.05; ### ≤ 0.001

To determine the bioavailability of our therapeutic oral 1,25D dose regime in mice, serum levels of 1,25D were tested 4 h after last 1,25D application. After oral gavage, serum 1,25D levels increased 6.1-fold in mice treated on three consecutive days with 10 ng 1,25D compared to control treatment (control: DMSO in peanut oil; Fig. 2a). For EAE experiments, we attempted to mimic GC pulse therapy of MS by treating the mice for three consecutive days starting at the onset of moderate signs of EAE. Mice first received doses of both substances (MP 0.8 mg/kg; 1,25D 10 ng) that had previously been demonstrated to only moderately improve symptoms of EAE [4, 53]. Whereas monotherapy with MP at this suboptimal dose did not lead to significant changes of clinical scores, 1,25D monotherapy resulted in mild clinical improvement compared to control treatment (control: PBS i.p. + DMSO in peanut oil p.o.; improvement of mean cumulative EAE score by 10.6%; *p* < 0.05, Fig. 2b). In contrast, strongest clinical effects were observed for the combination of 1,25D and MP (compared to control treatment reduction of mean cumulative EAE score by 19.1%, *p* < 0.001, Fig. 2b). To more closely resemble the situation in patients during MS relapse therapy, a higher GC dose (MP 4 mg/kg [37]) was used (Fig. 2c). Here, compared to control treatment (PBS i.p. + DMSO in peanut oil p.o.) respective monotherapies reduced the mean cumulative EAE score by 14.1% (MP 4 mg/kg) and 18.3% (1,25D 10 ng/day; each *p* < 0.001). Again, the strongest effect was observed for the MP/1,25D combination therapy, which ameliorated EAE disease score by 35.5% (*p* < 0.001; Fig. 2c). We next examined whether the synergistic effect of the 1,25D and MP combination therapy was dependent on the GR. In mice that are devoid of the GR in T cells (GR^lck^), this therapeutic combination regime did not lead to any clinical effects (Fig. 2d).

## 25D levels are reduced in MS patients with glucocorticoid-resistant relapses and T cell apoptosis is exclusively augmented by 1,25D in MS patients with glucocorticoid-resistant MS relapses

To address the potential relevance of our findings concerning the 1,25D/GC synergism in patients, we investigated 25D serum concentrations in patients with stable MS in comparison with patients with GC-responsive and GC-resistant MS relapses. Patient populations did not differ in gender or MS disease duration (Online Resource 2, initial cohort). Although GC-resistant patients were younger than those with GC-responsive MS relapses, age was not associated with 25D serum levels (*ρ* − 0.076, *p* > 0.05, *n* = 110). Baseline disability before relapse did not differ between groups (Online Resource 2). During relapse and before steroid administration, EDSS was higher in patients with GC-resistant compared to GC-responsive relapse. Although patients with GC-responsive relapse received approximately 3400 mg less methylprednisolone, only in these patients an EDSS improvement was observed. In contrast, patients with GC-resistant relapse even tended to further deteriorate despite high-dose GC treatment (Online Resource 2). In addition to the extent of improvement, patients with GC response also tended to improve more quickly after 0.5 (SD 0.6) months, whereas EDSS analysis in GC-resistant patients was performed after 0.7 (0.6) months (Online Resource 2). In GC-resistant patients with the longest interval between EDSS assessments post-relapse EDSS ≥ 1.5 months after first EDSS; *n* = 3) a prominent EDSS deterioration (+ 2.7 (2.3) was observed, arguing against delayed effects of GC.

**Fig. 3:**
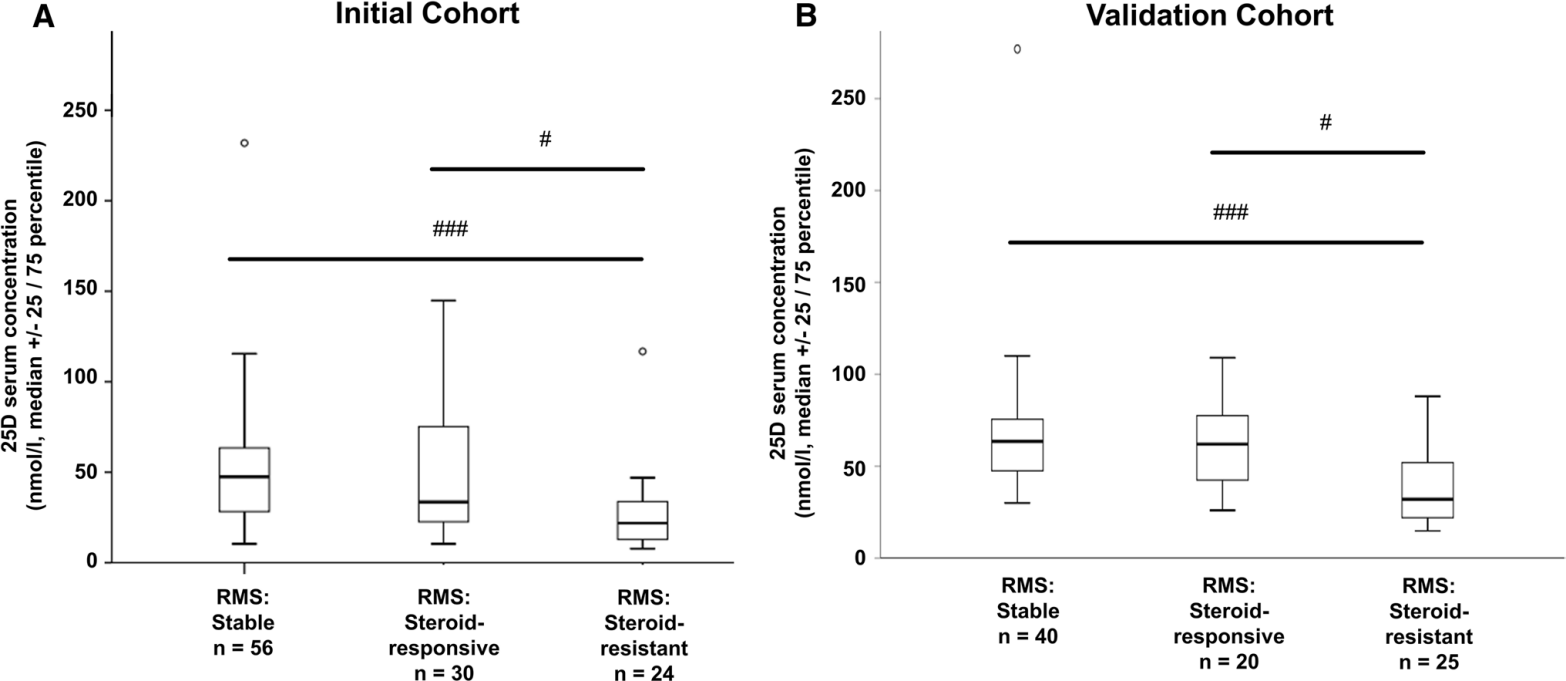
25D serum concentrations in nmol/L of patients with relapsing multiple sclerosis (RMS) in the initial cohort (**a**) and the validation cohort (**b**). Blood sampling was performed during stable disease (initial cohort: *n* = 56, validation cohort: *n* = 40), glucocorticoid-responsive relapse (validation cohort: *n* = 30, validation cohort: *n* = 20) or glucocorticoid-resistant relapse (initial cohort: *n* = 24, validation cohort: *n* = 25). *25D* 25-hydroxyvitamin D. Statistics: Kruskal–Wallis test: # < 0.05, ### ≤ 0.001

**Fig. 4:**
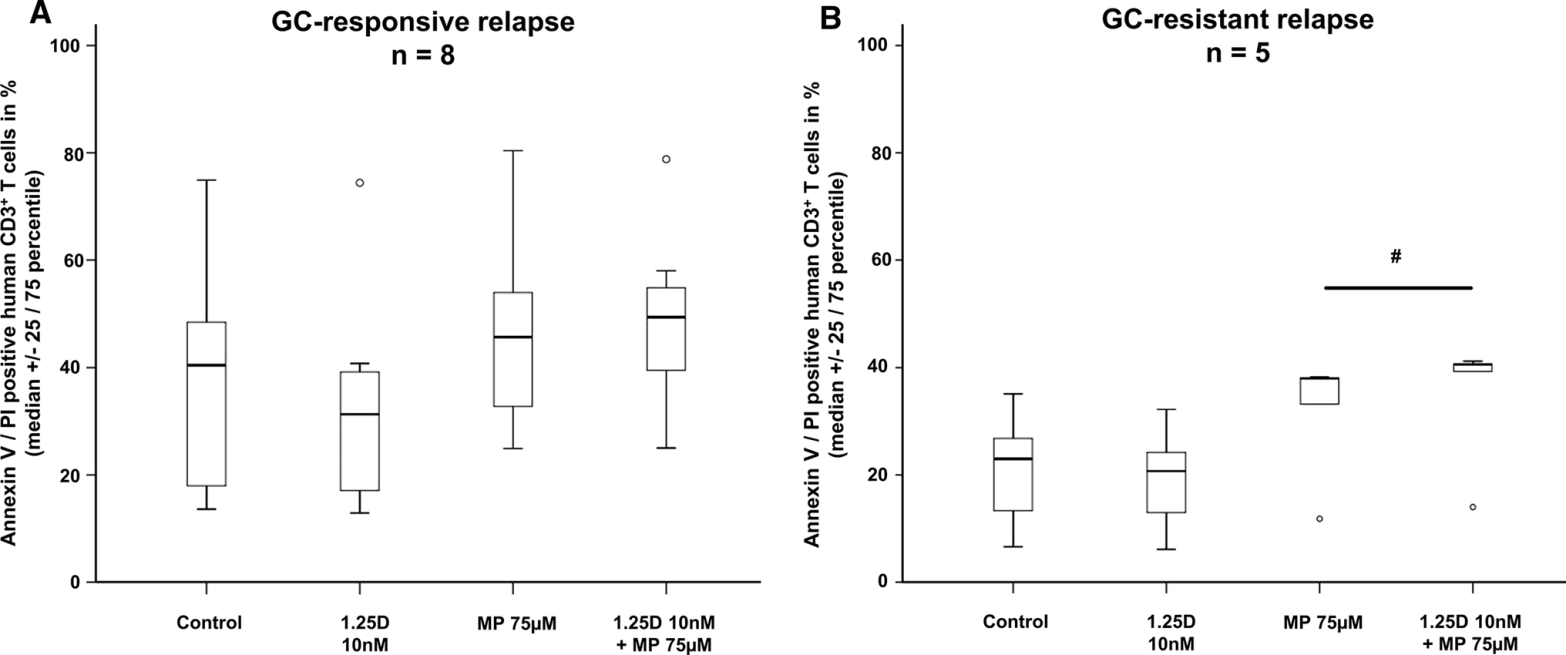
**a** Apoptosis of CD3^+^ T cells of MS patients during glucocorticoid-responsive relapse (*n* = 8) or **b** glucocorticoid-resistant relapse (*n* = 5). Incubation with control (solvent: 0.1% DMSO), 1,25D, MP or 1,25D + MP in vitro for 72 h (PHA 0.5 µg/ml; Annexin V/PI, flow cytometry). *GR* glucocorticoid receptor, *MP* methylprednisolone, *RMS* relapsing multiple sclerosis, *25D* 25-hydroxyvitamin D, *1,25D* 1,25(OH)_2_D_3_. Statistics: Wilcoxon signed-rank test: # < 0.05

Patients with GC-resistant relapse had polysymptomatic relapses with on average 0.4 more clinical symptoms than those who responded well to GC treatment. However, when analyzing distribution of relapse symptoms we did not observe specific patterns in patients with GC-resistant or GC-responsive relapse (e.g. optic neuritis vs. myelitis; Online Resource 3). Furthermore, patients with GC-resistant relapse exhibited on average 1.2 more gadolinium-enhancing lesions compared to those with GC-responsive relapse (Online Resource 2). Since GC-resistant patients had received higher GC dosages for relapse therapy, we investigated whether the GC dose influences 25D serum concentrations before and after GC treatment in a different subset of patients [median GC dosage 1665 mg (IQR 1525), min 750, max 7000, *n* = 10]. Here, no differences in 25D serum levels were observed before and after GC administration [median (IQR) 25D serum levels (nmol/l) prior to GC therapy: 7.2 (15.2) versus post GC pulse therapy: 10.0 (12.1), *p* > 0.05]. Overall, 25D serum levels in the initial cohort were almost 50% lower in MS patients with relapses than in stable patients [MS with relapses median (IQR): 27.6 nmol/l (25.7); stable MS without relapses 48.8 nmol/l (36.9); *p* < 0.01]. This difference was mainly due to the subgroup of patients with GC-resistant relapses. In these patients, 25D serum levels [21.9 (21.3) nmol/l] were 21% lower compared to GC-responsive MS (*p* < 0.05) and 55% lower compared to stable MS (*p* ≤ 0.001; Fig. 3a). A classification of 25D serum levels into established categories (< 25 nmol/l: severe deficiency; 25–49.9 nmol/l: deficiency; ≥ 50 nmol/l: sufficient 25D supply [18, 21]) demonstrated that 66.7% of GC-resistant MS patients were severely 25D deficient in contrast to only 26.7% of GC-responsive and 21.4% of stable MS patients. The influence of 25D serum concentration on GC efficacy was further validated using a multivariate logistic regression analysis adjusted for age, gender, MS disease duration, and presence of disease-modifying MS treatment. MS patients who experienced an acute relapse (*n* = 54) but did not exhibit severe 25D deficiency had a considerably higher likelihood of responding to GC pulse therapy than patients with severe 25D deficiency (odds ratio 10.6; 95% confidence interval 2.2–51.5, *p* < 0.05). Findings of reduced 25D serum concentrations in GC-resistant patients were validated using an independent cohort of RMS patients (validation cohort, *n* = 85, see Online Resource 4 for baseline characteristics). Also in this cohort, GC-resistant patients (*n* = 25) had lower 25D levels than stable (*n* = 40) and GC-responsive MS patients (*n* = 20, Fig. 3b). 1,25D values in this validation cohort did not demonstrate a correlation with 25D serum levels or a different distribution within the three MS patient groups (Online Resource 5). We next addressed whether the biologically active form of 25D, 1,25D, differentially affects GC-induced T cell apoptosis in GC-responsive (*n* = 8) and GC-resistant (*n* = 5) MS patients during relapse. Patients did not differ in gender, age, disease duration or the presence of immunotherapy (Online Resource 6). GC-resistant MS patients had received 2.7-fold higher dosage of GCs during GC pulse therapy (*p* < 0.05). Despite higher GC dosages, baseline apoptosis after GC therapy tended to be lower in these patients compared to patients with GC-responsive relapse (Fig. 4, *p* > 0.05). In GC-responsive MS patients, the addition of 1,25D (10 nM) did not lead to an additional effect of MP-induced T cell apoptosis in vitro (Fig. 4a). In contrast, 1,25D led to an increase of MP-induced apoptosis in T cells from GC-resistant MS patients (Fig. 4b, *p* < 0.05). To address if rather CD4^+^ or CD8^+^ T cells are involved, we investigated GR as well as HSP90-protein expression in blood-derived T cells from a limited group of RMS patients with GC-responsive and GC-resistant relapse by western blot. Here, a differential regulation of GR in CD8^+^ T cells was observed. Further a trend towards a GR regulation in CD4^+^ T cells as well as an increase in the HSP90/GR ratio in both T cell subsets was found (Online Resources 7, 8). In addition, active demyelinating MS lesions in brain biopsies of three patients (one with GC-resistant and two with GC-responsive relapse) were investigated to evaluate GR^+^ T cells in situ. Here, a pronounced infiltration of CD3^+^ as well as CD4^+^ and CD8^+^ T cells was present in the two patients with GC-responsive relapse compared to the single patient with GC-resistant relapse (Online Resources 9, 10). However, in this limited number of samples, no differences in GR expression were observed between phenotypes and between T cell subsets.

### Synergistic effects of 1,25D and glucocorticoids are mediated by inhibition of mTORc1 signaling

**Fig. 5:**
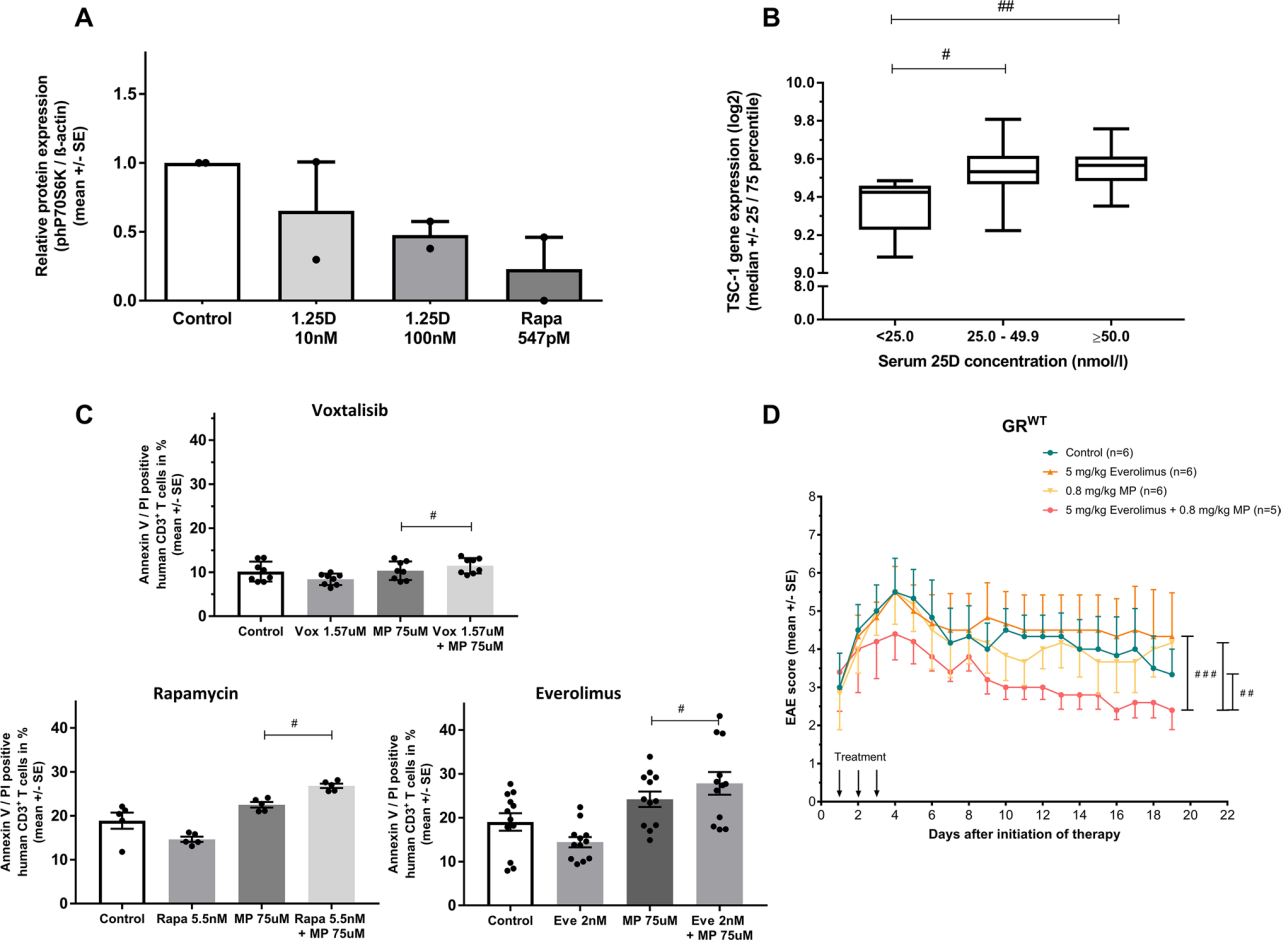
**a** Protein expression of phospho-P70 S6 Kinase (phP70S6K, downstream target of mTORc1) normalized to β-actin expression. Murine CD3^+^ T cells were incubated with control (solvent: 0.1% DMSO), 1,25D or rapamycin in vitro (24 h; ConA 1.5 µg/ml; *n* = 2 independent experiments, densitometry of western blots). **b** Log2 expression of TSC-1 gene in human CD8^+^ T cells in association with 25D serum concentrations (< 25 nmol/l; *n* = 5 (*n* = 2 healthy controls/*n* = 3 stable MS patients); 25–49.9 nmol/l; *n* = 42 (*n* = 24 healthy controls/*n* = 18 stable MS patients); ≥ 50 nmol/l; *n* = 65 (*n* = 37 healthy controls/*n* = 28 stable MS patients); see Online Resource 11; Affymetrix Human gene 1.0ST chip). **c** Apoptosis of human CD3^+^ T cells of healthy donors. Incubation with control (solvent: 0.1% DMSO), methylprednisolone and different mTOR inhibitors: voxtalisib (*n* = 4 in duplicates;) rapamycin (*n* = 5) and everolimus (*n* = 6 in duplicates) in vitro (24 h; PHA 0.5 µg/ml, Annexin V/PI, flow cytometry). **d** Disease course of MOG_35–55_ EAE in C57BL/6 wild type treated with control (solvent: DMSO in peanut oil), everolimus (5 mg/kg/day), MP (0.8 mg/kg/day) or everolimus + MP. Downwards arrow: days of treatment. EAE score: 10-score system according to Ref. [45]. Numbers of included animals are given in the figure. *MS* multiple sclerosis, *MP* methylprednisolone, *GR* glucocorticoid receptor, *phP70S6K* phospho-p70 S6 kinase, *Rapa* rapamycin, *SE* standard error, *TSC-1* tuberous sclerosis complex 1, *25D* 25-hydroxyvitamin D, *1,25D* 1,25(OH)_2_D_3_; *Vox* voxtalisib, Statistics: **b**, **d** Kruskal–Wallis test: # < 0.05; ## ≤ 0.01; **c** Wilcoxon signed-rank test: # < 0.05; ## ≤ 0.01

We next addressed if mTOR or JNK signaling pathways, both implicated in the regulation of GR expression, are also involved in the observed 1,25D/GC synergism. In murine T cells, 1,25D reduced protein expression of the mTORc1 downstream target phP70S6 K in a dose-dependent manner, proving the inhibition of mTORc1 activity (Fig. 5a). Likewise, gene expression of a key regulator of mTORc1 activity in human CD8^+^ T cells from a large cohort (Online Resource 11) was associated with 25D serum levels. Thus, expression of tuberous sclerosis complex (TSC)-1 was reduced in individuals with severe 25D deficiency (< 25 nmol/l; *n* = 5 (*n* = 2 healthy controls/*n* = 3 stable MS patients)) compared to individuals with 25D levels between 25–49.9 nmol/l (deficient; *n* = 42 (*n* = 24 healthy controls/*n* = 18 stable MS patients)) or with sufficient levels (≥ 50 nmol/l; *n* = 65 (*n* = 37 healthy controls/*n* = 28 stable MS patients); Fig. 5b). No associations between 25D serum levels and Akt, TSC-2 or Rheb gene expression were observed (data not shown). We next demonstrated that three different types of mTOR inhibitors increased GC-induced apoptosis in human T cells (Fig. 5c), indicating a class-specific effect. In contrast, GC co-incubation with SP600125, a pharmacological JNK inhibitor [5], did not alter GC-induced human T cell apoptosis (Online Resource 12A) and had no therapeutic effect on GC treatment efficacy in EAE (Online Resource 12B).

**Fig. 6:**
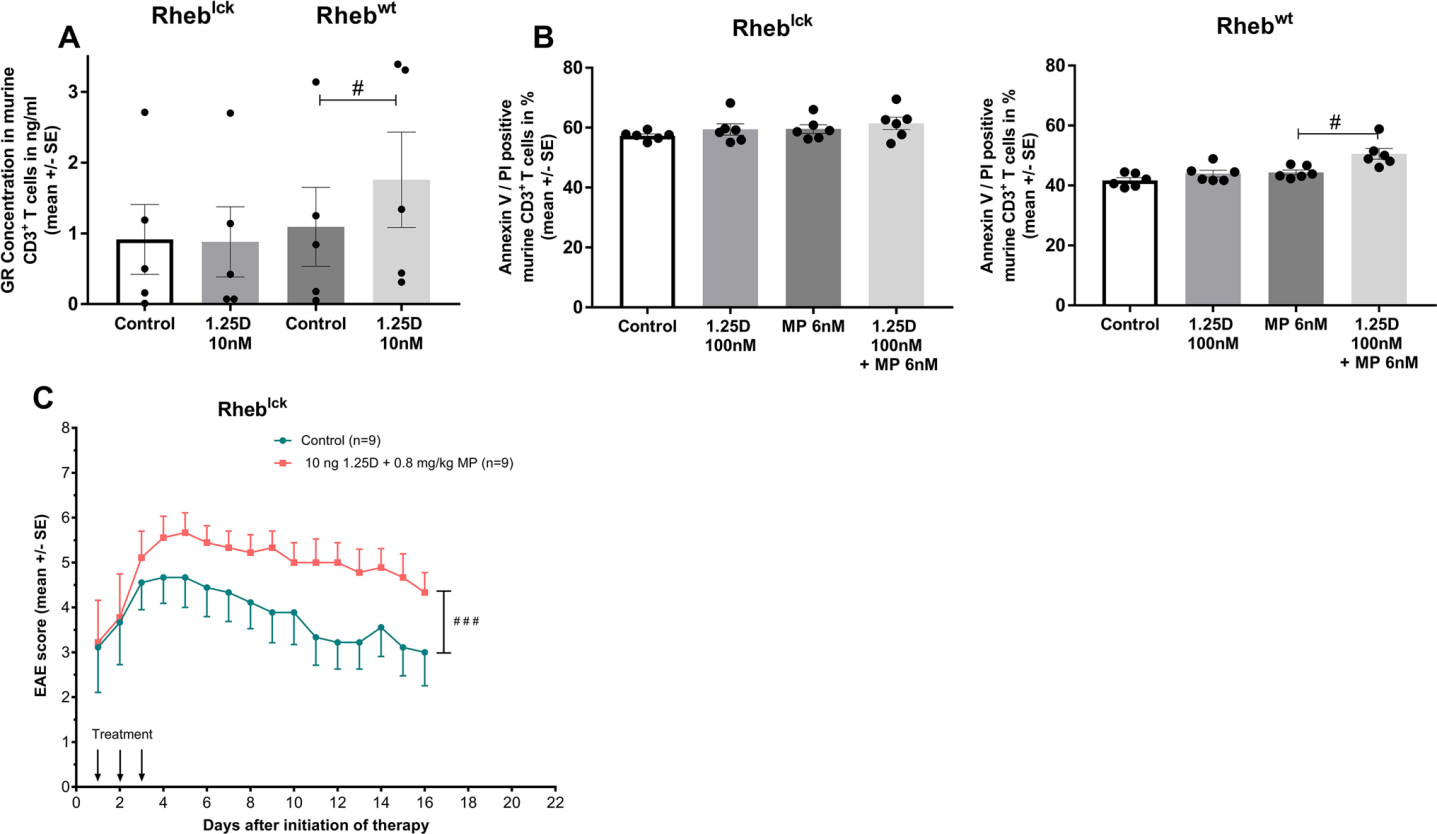
**a** GR protein concentration in ng/ml after incubation of murine CD3^+^ T cells (Rheb^lck^ vs. Rheb^wt^) with 1,25D in vitro (24 h; ConA 1.5 µg/ml, *n* = 5; ELISA). **b** Apoptosis of murine splenic-derived CD3^+^ T cells from C57BL/6 Rheb^wt^ or Rheb^lck^ mice. Incubation with control (solvent: 0.1% DMSO), 1,25D 100 nM, MP 6 nM or 1,25D + MP in vitro (24 h; ConA 1.5 µg/ml; *n* = 3 in duplicates; Annexin V/PI, flow cytometry). **c** Disease course of MOG_35–55_ EAE in Rheb^lck^ treated for three consecutive days with control (solvent: DMSO in peanut oil) or 1,25D (10 ng/day) + MP (0.8 mg/kg/day). Downwards arrow: days of treatment. EAE score: 10-score system according to Ref. [45]. Numbers of included animals are given in the figure. *GR* glucocorticosteroid receptor, *MP* methylprednisolone, *Rheb* ras homolog enriched in brain, *SE* standard error, *1,25D* 1,25(OH)_2_D_3_, *95% CI* 95% confidence interval. Statistics: **a**, **b** Wilcoxon signed-rank test: # < 0.05; **c**, **d** Kruskal–Wallis test: ## ≤ 0.01, ### ≤ 0.001

To further investigate the relevance of the mTORc1 pathway for 1,25D/GC synergism, we used murine T cells deficient in mTORc1 signaling (Rheb^lck^). In these cells, 1,25D failed to upregulate GR and to increase GC-induced apoptosis (Fig. 6a, b). Further, in EAE, combined 1,25D/GC treatment in Rheb^lck^ mice worsened clinical disease course in comparison with control treatment (control: PBS i.p. + DMSO in peanut oil p.o.; Fig. 6c). We next assessed the impact of pharmacological mTORc1 inhibition on EAE using everolimus, a rapalog, which allosterically and specifically blocks mTORc1 activity [10]. Everolimus was administered in a dose (5 mg/kg, p.o.) that was demonstrated to result in a strong mTORc1 inhibition in vivo [11]. Whereas respective monotherapies did not have any relevant effect on clinical EAE disease course compared to control treatment (control: PBS i.p. + DMSO in peanut oil p.o.), the combination therapy with everolimus and MP led to a decrease of EAE disease severity by 23.5% compared to control (Fig. 5d, *p* < 0.01).

## Discussion

Given the central role of the GR in mediating clinical GC resistance [38], we investigated if VD augments GC efficacy via modulation of GR expression [39].

We found that 1,25D increased GR protein levels and enhanced GC responsiveness in vitro and in vivo. Synergistic efficacy of the 1,25D/GC combination therapy was dependent on the GR in T cells as demonstrated using two different mouse models with reduced or absent GR signaling. Low 25D serum levels were associated with decreased GR mRNA expression in human T cells and MS patients with profound 25D deficiency were more likely to exhibit clinical resistance to GCs during relapse. Likewise, 1,25D increased GC effects in vitro only in T cells from GC-resistant MS patients. Several findings point to a crucial role of mTORc1 signaling in mediating 1,25D/GC functional synergism. Severe 25D deficiency was associated with a downregulation of an archetype mTORc1 inhibitor in human T cells. In animals with T cell-specific depletion of mTORc1, the synergistic 1,25D/GC effects on GR upregulation, T cell apoptosis and therapeutic efficacy in EAE were abolished. Finally, treatment with a specific mTORc1 inhibitor also augmented therapeutic GC efficacy in EAE.

An association of hypovitaminosis D (25D-deficiency) and increased use of steroids was described for other inflammatory diseases such as asthma and inflammatory bowel disease [20, 47], and different effector cells and mechanisms have been implied in mediating this effect. In EAE, T cells appear to be pivotal in mediating synergistic effects of 1,25D and GC, since T cell-specific deletion of either GR or mTORc1 abrogated these effects. This fits well with previous data demonstrating lack of efficacy of GC monotherapy in EAE only when the GR is deleted in T cells, but not in monocytes, macrophages and microglia [54]. Upregulation of the GR has been associated with a better prognosis and increased clinical efficacy of GC treatment in several immune-mediated and malignant diseases [28, 50]. It is noteworthy that the GR can either regulate gene transcription through binding to GC-responsive elements (GRE) presented in regulatory DNA regions, or by interacting with transcription factors such as nuclear factor kappa B or activator protein-1 [16]. In GR^dim^ mice, dimerization of the GR and thus its ability to bind to GREs are strongly impaired. As a consequence, regulation of at least a subset of genes by the GR is abolished [37]. The lack of any 1,25D/GC synergism in GR^dim^ cells, therefore, argues that altered gene expression is relevant for the observed effects being in line with the finding of increased GR binding to a GRE after pre-incubation with VD [57]. We demonstrated clear synergism of 1,25D with MP as the evidence-based GC for the treatment of acute MS relapse. As the synergism is mediated via an upregulation of the GR, it appears likely that this effect is not specific for MP and might be used to increase efficacy of other GCs, e.g. dexamethasone. In vitro, VD/dexamethasone combination suppresses the activation of synovial fibroblasts via modulation of CCR6^+^ T-helper memory cells [8]. We did not observe any clinical effect of 1,25D monotherapy in mice with a T cell-specific GR-deficiency, in contrast to robust efficacy in WT mice, in line with the well-known VD effects on MOG_35–55_ EAE [7, 56]. Since the VD receptor and GR are located on different chromosomes (VDR: 12q13.11; GR: 5q31.3 [32]), a coincidental knock-out of both receptors by the Cre recombinase expressed in GR^lck^ T cells appears unlikely. Therefore, we speculate that the GR might also be relevant for specific immunological effects of VD, a hypothesis that warrants further investigation.

For the analysis of systemic VD supply in our MS patient cohorts, the major circulating metabolite (25D) was used because it has a longer half-life than bioactive 1,25D and is less influenced by hormones or medications [24]. Reduced 25D serum levels in patients with GC-resistant relapse compared to stable patients and patients with GC-responsive relapse were observed in two independent cohorts of RMS patients. Lack of correlation of 1,25D with 25D serum levels points to higher sensitivity of 25D serum levels, since 1,25D serum concentration is kept in the normal range even if 25D concentration is reduced [24]. In addition, systemic 1,25D serum levels will not properly reflect local bioactive 1,25D, since T cells and macrophages also have the ability to directly convert 25D into 1,25D at the site of action, which cannot be detected by analyzing serum samples [36]. Together, these factors argue for the use of 25D serum concentration as a global measure of the body’s VD supply as is performed in most epidemiological and interventional studies [3, 35].

In our multivariate analysis, corrected for various potential confounders (age, gender, MS disease duration, presence of disease-modifying MS treatment), severe 25D deficiency was associated with clinical GC resistance even after GC dose escalation. The clinical definition of GC resistance followed current national guidelines on the use of plasma exchange as evidence-based treatment intensification for MS relapses [13]. The retrospective data analysis in our patient cohorts is a clear limitation. Although we did not observe major clinical or epidemiological characteristics between the GC-responsive and -resistant patients that confounded our findings to a large extent, these cannot completely be excluded. Especially, masking of delayed GC effects due to early initiation of plasma exchange as intensified relapse treatment according to national guidelines cannot completely be ruled out [13]. However, in a more recent prospective double-blinded study, an EDSS improvement already after 28 days was demonstrated which did not further change after 180 days [22]. In addition, severely reduced 25D levels in GC-resistant patients in two independent cohorts also argue for robustness of our findings.

Also, our experimental data on the synergistic effect of 1,25D on GC-induced apoptosis exclusively in T cells from GC-resistant patients argue for a true biological difference between the cohorts.

In patients with GC-resistant relapse, basal T cell apoptosis ex vivo was lower compared to steroid-responsive MS patients despite application of twofold higher GC dosages in steroid-resistant patients. This observation is in line with previous data demonstrating lower suppressive capacity of dexamethasone on T cell proliferation in GC-resistant than in GC-responsive patients [29]. Peripheral CD8^+^ T cells from a limited number of patients with GC-resistant relapse had a higher GR protein expression in comparison with cells from patients with GC-responsive relapse, similar to previous findings in peripheral mononuclear cells [29]. In early active demyelinating CNS lesions, in one investigated GC-resistant patient there were less T cells, albeit the expression of the GR did not appear to differ between patients with and without GC response. These preliminary findings will have to be followed up also with respect to the presumed site of action of GC.

Concerning the signaling mechanism for 1,25D/GC synergism, we identified a role of the mTORc1 but not the JNK pathway downstream of VD. Both genetic deletion and pharmacological inhibition point to mTORc1 inhibition as major mechanism mediating 1,25D/GC synergism. In individuals with severe 25D-deficiency TSC-1 gene expression was reduced. Since TSC-1 is an archetype mTORc1 inhibitor [19], downregulation of TSC-1 would result in a disinhibition of the mTORc1 pathway. Furthermore, our in vitro data indicated a functional inhibition of mTORc1 enzyme activity by 1,25D in T cells, with inhibition of phosphorylation of a downstream target of mTORc1.

In vitro, T cells from Rheb^lck^ mice have a T cell-specific depletion of mTORc1 activity [9]. In Rheb-deficient T cells, 1,25D did not lead to GR upregulation and these mice did not exhibit synergistic 1,25D/GC effects in apoptosis induction. Moreover, EAE data in Rheb^lck^ animals also supported our conclusion, as we did not find any inhibitory effect on the disease by the 1,25D/GC combination. Similar to findings reported before also in our hands active MOG_35–55_ EAE resulted in a more cerebellar phenotype, which is presumably associated with a more pronounced Th2 rather than Th1/Th17 CNS autoimmunity [9]. VD and GCs drive Th2 polarization [12, 48], which could hypothetically explain the exacerbated disease course of MOG_35–55_ EAE in Rheb^lck^ mice treated with the combination of 1,25D/GC. Finally, synergistic effects of everolimus and GC in WT EAE mice point to mTORc1-inhibition as major mechanism responsible for the increased GC efficacy. Everolimus, a derivative of rapamycin, is a potent mTORc1 inhibitor and approved for the treatment of tuberous sclerosis [49]. Thus, rapid translation into the clinical setting of MS relapse treatment appears feasible, especially since its usage would be temporally restricted, potentially reducing possible adverse drug reactions (e.g. infections and ulcera).

Despite highly efficacious disease-modifying therapies, GCs remain the most commonly used substances in MS treatment [17, 40]. Nevertheless, approximately 30% of MS patients report insufficient remission and 50% suffer from steroid-related side effects [33]. The use of plasma exchange/immune adsorption as treatment intensification during acute relapse constitutes an invasive procedure. Thus, further translation of alternative approaches to improve treatment of acute neuroinflammation would meet a high clinical need. However, given the high heterogeneity of relapse phenotypes and dynamics, clinical studies face several hurdles, e.g. sample size and sensitive, objective readouts. Acute optic neuritis may represent an interesting paradigm where biologically meaningful outcome parameters can be objectively and sensitively quantified [34]. Finally, our results may also harbor implications for other clinical situations, since GCs are widely used in the treatment of autoimmune disease and malignancies.

## Electronic supplementary material

Below is the link to the electronic supplementary material.

Supplementary material 1 (DOCX 64 kb)

Supplementary material 2 (JPG 2012 kb)

Supplementary material 3 (JPG 2098 kb)

Supplementary material 4 (JPG 2077 kb)

Supplementary material 5 (JPG 2660 kb)

Supplementary material 6 (JPG 2574 kb)

Supplementary material 7 (JPG 2331 kb)

Supplementary material 8 (XLSX 68 kb)
