# Supplementary material for: Vitamin D increases glucocorticoid efficacy via inhibition of mTORC1 in experimental models of multiple sclerosis
Source: Acta Neuropathol. 2019 Apr 27;138(3):443–56. doi: 10.1007/s00401-019-02018-8 (PMC6689294; doi:10.1007/s00401-019-02018-8)
Supplement: Supplementary file 1 — Supplementary material 1 (DOCX 64 kb) [file 401_2019_2018_MOESM1_ESM.docx]

*Online Resources*

*Online Resource 1:* Apoptosis of murine splenic-derived CD3^+^ T cells from C57BL/6 wild type mice. Mice were fed over three consecutive days with 1.25D (20ng per day, p.o.) or control (DMSO in peanut oil, p.o.). Splenic CD3^+^ T cells were incubated with MP (6nM, 60nM, 600nM) or control (solvent of MP: PBS) *in vitro* (24h; n = 5; ConA 1.5µg/ml; Annexin V / PI, flow cytometry). *Abbreviations:* MP: methylprednisolone; SE: standard error; 1.25D: 1.25(OH)_2_D_3_. *Statistics:* Mann Whitney Test: ## < 0.01.

*Online Resource 2:* Baseline characteristics of relapsing MS patient groups with stable disease, glucocorticosteroid responsive and glucocorticosteroid resistant relapse (initial cohort).

|  | RMS: stable  (n=56) | RMS: steroid responsive relapse (n=30) | RMS: steroid resistant relapse (n=24) | p-value |
| --- | --- | --- | --- | --- |
| Gender (female) | 40/56 (71.4 %) | 24/30 (80.0 %) | 15/24 (62.5 %) | n.s. |
| Age (years; mean (SD)) | 39.1 (13.8) | 40.3 (11.7) | 32.2 (9.5) | stable vs. resistant <0.05 |
| MS disease duration (years; mean (SD)) | 7.4 (6.4) | 6.1 (6.9) | 6.2 (7.6) | n.s. |
| Dose GC (mg; mean (SD)) | 0.18 (1.3) | 3973.3 (2830.2) | 7353.3 (4159.3) | stable vs. responsive ≤0.001  stable vs. resistant ≤0.001 |
| Number of Gadolinium enhancing lesions (mean (SD)) | N/A | 1.0 (1.6)^3^ | 2.2 (1.9)^7^ | resistant vs responsive <0.05 |
| 25D serum concentration (nmol/l; median (25^th^/75^th^ percentile)) | 48.8 (28.7 - 65.5) | 33.5 (22.0 - 75.7) | 21.9 (12.7 – 34.0) | stable vs. responsive <0.05  stable vs. resistant ≤0.001 |
| EDSS during stable disease or before relapse (mean (SD)) | 2.8 (1.9)^1^ | 1.8 (1.8)^5^ | 2.5 (2.8)^3^ | n.s. |
| EDSS during relapse before glucocorticosteroids (mean (SD)) | N/A | 3.3 (1.6)^1^ | 4.7 (2.1) | resistant vs responsive 0.009 |
| EDSS after glucocorticosteroids (mean (SD)) | N/A | 2.3 (1.8) | 5.1 (2.2)^1^ | resistant vs responsive <0.001 |
| Time between EDSS assessments (months; mean (SD)) | N/A | 0.5 (0.6)^1^ | 0.7 (0.6)^1^ | n.s. |
| ARR in the year prior to relapse or sampling (mean (SD)) | 0.2 (0.5)^2^ | 1.2 (0.4) | 1.4 (0.7) | stable vs. responsive <0.001  stable vs. resistant <0.001 |
| ARR in the year after relapse or sampling (mean (SD)) | 0.1 (0.3)^6^ | 0.2 (0.5)^7^ | 0.3 (0.5)^7^ | n.s. |
| Isolated optic neuritis | N/A | 4/30 | 7/24 | n.s. |
| Isolated myelitis | N/A | 4/30 | 1/24 | n.s. |
| No immunotherapy at 25D sampling or at relapse | 10/56 (17.9 %) | 14/30 (46.7 %) | 16/24 (66.7 %) | stable vs. responsive ≤0.01  stable vs. resistant ≤0.001 |
| Teriflunomide | 2/56 | 1/30 | 0/24 |  |
| Copaxone | 8/56 | 4/30 | 0/24 |  |
| Interferon | 12/56 | 4/30 | 3/24 |  |
| Dimethyl Fumarate | 13/56 | 2/30 | 3/24 |  |
| Fingolimod | 8/56 | 4/30 | 2/24 |  |
| Natalizumab | 1/56 | 1/30 | 0/24 |  |
| Anti-CD20 | 2/56 | 0/30 | 0/24 |  |

*Abbreviations:* ARR: Annualized relapse rate; EDSS: Expanded Disability Status Scale, GC: glucocorticoid, N/A: not applicable, n.s.: not significant, RMS: relapsing multiple sclerosis, SD: standard deviation, 25D: 25-hydroxyvitamin D. *Statistics:* Chi² test was used to compare categorical and Kruskal-Wallis Test for comparison of continuous data. ^1^ One missing values; ^2^ Two missing values, ^3^ Five missing values, ^4^ Seven missing values, ^5^ Eight missing values, ^6^ Ten missing values, ^7^ Eleven missing values. Index relapse was included in the ARR one year prior to relapse.

*Online Resource 3:* Relapse symptoms of relapsing MS patients with *(A)* GC-responsive and *(B)* GC-resistant relapse. *Abbreviations:* GC: glucocorticosteroid.

*Online Resource 4:* Baseline characteristics of relapsing MS patient groups (validation cohort) with stable disease, GC-responsive and GC-resistant relapse.

|  | RMS: stable  (n=40) | RMS: steroid responsive relapse (n=20) | RMS: steroid resistant relapse (n=25) | p-value |
| --- | --- | --- | --- | --- |
| Gender (female) | 27/40 | 14/20 | 18/25 | n.s. |
| Age (years; mean (SD)) | 37.9 (13.6) | 35.2 (11.6) | 37.0 (8.8) | n.s. |
| Age at MS diagnosis (years; mean (SD)) | 28.9 (11.4)^1^ | 30.1 (11.4)^2^ | 32.7 (8.0)^1^ | n.s. |
| Dose GC (mg; mean (SD)) | N/A | 2818.0 (1129.7) | 6108 (2979.1) | responsive vs resistant <0.001 |
| 25D serum concentration (nmol/l; median (25^th^/75^th^ percentile) | 63.5 (47.3 – 76.8) | 62.0  (42.3 – 77.8) | 32.0  (20.7 – 58.5) | stable vs resistant 0.0004  responsive vs resistant 0.017 |
| 1.25D serum concentration (pmol/l; median (25^th^/75^th^ percentile) | 111.0 (75.0 – 143.3) | 129.5  (77.3 – 163.5) | 113.5  (75.0 – 795.3)^3^ | n.s. |
| EDSS during stable disease or during relapse before glucocorticosteroids (mean (SD)) | 2.2 (1.5)^4^ | 3.3 (1.7)^3^ | 3.3 (1.6) | resistant vs stable 0.006  responsive vs stable 0.045 |
| EDSS after glucocorticosteroids (mean (SD)) | N/A | 2.3 (1.6)^1^ | 3.4 (1.4) | responsive vs resistant 0.04 |
| Time between EDSS assessments (months; mean (SD)) | N/A | 1.3 (1.3)^1^ | 0.8 (1.1) | responsive vs resistant 0.04 |
| No immunotherapy at 25D sampling or at relapse | 4/40 | 13/20 | 16/25 | resistant vs stable <0.001  responsive vs stable <0.001 |
| Copaxone | 6/40 | 2/20 | 1/25 |  |
| Interferon | 2/40 | 2/20 | 1/25 |  |
| Dimethyl Fumarate | 9/40 | 1/20 | 3/25 |  |
| Fingolimod | 7/40 | 1/20 | 1/25 |  |
| Natalizumab | 7/40 | 1/20 | 1/25 |  |
| Anti-CD20 | 5/40 | 0/20 | 2/25 |  |

*Abbreviations:* EDSS: Expanded Disability Status Scale, GC: glucocorticoid, N/A: not applicable, n.s.: not significant, RMS: relapsing multiple sclerosis, SD: standard deviation, 25D: 25-hydroxyvitamin D, 1.25D: 1,25(OH)_2_D_3_. *Statistics:* Chi² test was used to compare categorical and Kruskal-Wallis Test for comparison of continuous data. ^1^ One value missing, ^2^ Two values missing, ^3^ Three values missing, ^4^ Five values missing.

*Online Resource 5:* *(A)* Association between 25D and 1.25D serum concentrations in RMS patients with different disease courses (validation cohort with available 25D and 1.25D measurements, n = 82). *Statistics:* Pearson correlation coefficient -0.042, p = 0.71. *(B)* 1.25D serum concentration in pmol/L of patients with relapsing multiple sclerosis (RMS, validation cohort) with stable disease (n = 40), steroid-responsive (n = 20) as well as steroid-resistant relapse (n = 22). *Statistics:* Kruskal-Wallis Test, p = 0.61. *Abbreviations:* RMS: relapsing multiple sclerosis, 25D: 25-hydroxyvitamin D, 1.25D: 1,25(OH)_2_D_3_.

*Online Resource 6:* Baseline characteristics of MS patient groups with steroid responsive or resistant relapse included in the *in vitro* analysis of GC-induced T cell apoptosis.

|  | RMS: steroid responsive relapse (n=8) | RMS: steroid  resistant relapse (n=5) | p-value |
| --- | --- | --- | --- |
| Gender (female) | 6/8 (75 %) | 5/5 (100 %) | n.s. |
| Age in years (mean (SD)) | 35.9 (13.4) | 36.8 (10.1) | n.s. |
| MS disease duration in years (mean (SD)) | 8.1 (8.8) | 2.8 (3.0) | n.s. |
| No immunotherapy at 25D sampling | 3/8 (37.5 %) | 3/5 (60 %) | n.s. |
| Dose GC in mg (mean (SD)) | 2837.5 (2364.4) | 7800 (3701.4) | 0.02 |

*Abbreviations:* GC: glucocorticoid; n.s.: not significant; RMS: relapsing multiple sclerosis; SD: standard deviation; 25D: 25-hydroxyvitamin D. *Statistics:* Chi² test was used to compare categorical and Mann Whitney Test for comparison of continuous data.

*Online Resource 7: (A)* Glucocorticoid receptor and *(B)* HSP90 protein expression normalized to GAPDH as well as (C) HSP90/GR ratio in CD4^+^ and CD8^+^ human T cells of RMS patients with GC-responsive (n = 4) and GC-resistant (n = 7) relapse. *Abbreviations:* GAPDH: Glycerinaldehyd-3-phosphat-Dehydrogenase; GR: Glucocorticoid Receptor; HSP90: heat shock protein 90, SE: Standard Error; *Statistics:* one-sided Mann Whitney Test [29]: # < 0.05.

*Online Resource 8:* Patient characteristics of patients with western blot analyses (Online Resource 7)

|  | **GC-resistant** | | **GC-responsive** | |
| --- | --- | --- | --- | --- |
|  | **CD4** | **CD8** | **CD4** | **CD8** |
| Gender (female) | 5/7 | 5/7 | 2/4 | 1/4 |
| Age (years; mean (SD)) | 35.6 (11.6) | 35.6 (11.6) | 33.0 (8.0) | 35.8 (4.7) |
| Age at RMS diagnosis (years; mean (SD)) | 32.6 (13.1) | 32.6 (13.1) | 32.0 (6.7) | 34.8 (3.0) |
| No immunotherapy at 25D sampling | 4/7 | 4/7 | 3/4 | 3/4 |
| Dose GC (mg; mean (SD)) | 6071 (3769) | 6071 (3769) | 5500 (3000) | 4500 (1915) |
| EDSS during relapse before glucocorticosteroids (mean (SD) | 2.5 (1.2) | 2.5 (1.2) | 2.6 (0.5) | 2.6 (0.5) |
| EDSS after glucocorticosteroids (mean (SD)) | 3 (1.6) | 3 (1.6) | 1.5 (0.4) | 1.6 (0.3) |
| Time between EDSS assessments (months; mean (SD)) | 25.1 (29.0) | 25.1 (29.0) | 15.5 (10.1) | 12 (3.9) |
| No Immunotherapy | 4/7 | 4/7 | 3/4 | 3/4 |

*Abbreviations:* EDSS: Expanded Disability Status Scale, GC: glucocorticoid, RMS: relapsing multiple sclerosis, SD: standard deviation, 25D: 25-hydroxyvitamin D, 1.25D: 1,25(OH)2D3.

*Online Resource 9:* Patient characteristics of the three biopsied patients (Online Resource 10)

| GC response | Course | Age (y) | Gender | Site of biopsy | Time from relapse onset to biopsy (days) | EDSS prior GC | EDSS post GC | CD3^+^  (mm^2^) | % GR^+^ CD3^+^ | CD4^+^  (mm^2^) | % GR^+^ CD4^+^ | CD8^+^ (mm^2^) | % GR^+^ CD8^+^ |
| --- | --- | --- | --- | --- | --- | --- | --- | --- | --- | --- | --- | --- | --- |
| No | Monophasic | 55 | M | Left frontoparietal | 12 | 4.5 | 4.5 | 64 | 100 | 38 | 71 | 36 | 100 |
| Yes | RMS | 45 | F | Right frontal | 18 | 3.0 | 1.5 | 1746 | 93 | 716 | 65 | 537 | 81 |
| Yes | Monophasic | 32 | F | Left parietofrontal | 18 | 2.5 | 0.0 | 677 | 95 | 257 | 100 | 178 | 100 |

*Abbreviations:* EDSS: Expanded Disability Status Scale, F: Female, GC: Glucocorticosteroid, M: Male, RMS: Relapsing MS, Y: Years. *Comment:* GC dose is unknown.

*Online Resource 10:* Costaining of the glucocorticoid receptor (GR, red) with either CD3 (green), CD4 (green) or CD8 (green) in human early active demyelinating MS-lesions from patients with either glucocorticosteroid-sensitive or -resistant relapse. Stereotactic biopsies were taken prior to GC-administration.

*Online Resource 11:* Baseline characteristics of RMS patients and healthy controls included in the gene expression analysis of tuberous sclerosis complex-1 (TSC-1) and GR.

|  | All Patients (n=112) | Untreated RMS Patients (n=49) | Healthy Controls (n=63) | P-value (RMS vs. Healthy) |
| --- | --- | --- | --- | --- |
| Gender (female) | 73/112 (65.2 %) | 36/49 (73.5 %) | 37/63 (58.7 %) | n.s. |
| Age (years; mean (SD)) | 37.1 (9.7) | 38.3 (9.7) | 36.2 (9.8) | n.s. |
| MS disease duration (years; mean (SD)) | N/A | 8.8 (7.8) | N/A | N/A |
| No Immunotherapy at 25D sampling | 112/112 (100 %) | 49/49 (100 %) | 63/63 (100 %) | n.s. |
| EDSS score at sampling (mean (SD)) | N/A | 1.9 (1.7) | N/A | N/A |
| 25D serum concentration (nmol/l; median (25^th^/75^th^ percentile)) | 56.50  (41.25 – 78.50) | 54  (39.00 – 76.50) | 58  (43.00 – 79.00) | n.s. |

*Abbreviations:* EDSS: Expanded Disability Status Scale; GR: Glucocorticoid receptor; RMS: relapsing multiple sclerosis; N/A: not adjusted; n.s.: not significant; 25D: 25-hydroxyvitamin D*. Statistics:* Chi² test was used to compare categorical and Mann Whitney Test for comparison of continuous data.

*Online Resource 12: (A)* Apoptosis of human CD3^+^ T cells of healthy donors. Incubation with control (solvent: 0.1% DMSO), SP600125 (SP, JNK inhibitor, 10nM), methylprednisolone (75µM) and SP + MP *in vitro* (72h; PHA 0.5µg/ml; n = 5 per group; Annexin V / PI, flow cytometry). *(B)* Disease course of MOG_35-55_ EAE in C57BL/6 wild type treated with control (solvent: DMSO in peanut oil), SP600125 (SP, 15mg/kg per day), MP (0.8mg/kg per day) or SP + MP. ↓ Days of treatment. EAE score: 10 score system according to [45]. Numbers of included animals are given in the figure. *Abbreviations:* MP: methylprednisolone, Rapa: rapamycin, SE: standard error, SP: SP600125. *Statistics:* (A) Wilcoxon signed-rank test: # < 0.05; ## ≤ 0.01. (B) Kruskal-Wallis Test: ### ≤ 0.001.
